# Tumor Antigen Cross-Presentation and the Dendritic Cell: Where it All Begins?

**DOI:** 10.1155/2010/539519

**Published:** 2010-10-13

**Authors:** Alison M. McDonnell, Bruce W. S. Robinson, Andrew J. Currie

**Affiliations:** ^1^School of Medicine and Pharmacology, University of Western Australia, Perth, WA 6009, Australia; ^2^National Centre for Asbestos Related Diseases, Perth, WA 6009, Australia; ^3^School of Veterinary and Biomedical Sciences, Murdoch University, WA 6150, Australia

## Abstract

Dendritic cells (DCs) are professional antigen-presenting cells (APCs) that are critical for the generation of effective cytotoxic T lymphocyte (CTL) responses; however, their function and phenotype are often defective or altered in tumor-bearing hosts, which may limit their capacity to mount an effective tumor-specific CTL response. In particular, the manner in which exogenous tumor antigens are acquired, processed, and cross-presented to CD8 T cells by DCs in tumor-bearing hosts is not well understood, but may have a profound effect on antitumor immunity. In this paper, we have examined the role of DCs in the cross-presentation of tumor antigen in terms of their subset, function, migration, and location with the intention of examining the early processes that contribute to the development of an ineffective anti-tumor immune response.

## 1. Introduction

MHC class I-restricted tumor antigens derived from peripheral solid tumors can be presented by host APCs to naïve CD8^+^ T cells in a process known as cross-presentation. Though the contribution of this pathway to the generation of antitumor immunity has been challenged [1, 2], there is convincing evidence that tumor antigens are efficiently cross-presented *in vivo *[3–8]. Despite this, the coexistence of an antitumor immune response with tumor progression suggests that tumor-specific CD8^+^ T cells are not properly activated *in vivo* or that tumor evasion mechanisms operate. Indeed, many have suggested that the inherent response of the host immune system to the cross-presentation of tumor antigens is the induction of T-cell tolerance [9–13]. Several studies have shown that incomplete activation of CD8^+^ T cells in the tumor draining lymph node (TDLNs) is due to the effect of the tumor on dendritic cells (DCs), which are thought to be the major cross-presenting APC, rather than on the T cells themselves [5, 8, 10, 14–16]. Indeed, DCs detected in tumor tissue or local tumor draining lymph nodes (TDLN) of cancer patients display an immature phenotype defined by low expression of CD80, CD86 or CD83, and altered APC function [5, 15, 17–19]. Since CD8^+^ T cells are critical for the surveillance, control, and rejection of tumors [20, 21], an understanding of how existing tumor-specific responses are initially generated, and how this leads to their dysfunction, may ultimately lead to improved immunotherapy's for cancer.

## 2. Mechanism of Cross-Presentation

The process of cross-presentation was first identified by Bevan and colleagues in the mid-1970s where they showed that immunization with lymphoid cells congenic for minor histocompatibility antigens resulted in the generation of CD8^+^ T cells that were restricted by the host MHC class I molecules [22, 23]. Thus, minor histocompatibility antigens must have been transferred from the donor cells to host APCs for priming of CD8^+^ T cells [22, 23]. Whilst the exact mechanisms allowing exogenous antigen into the MHC class I processing pathway have not been fully elucidated, two main pathways have been described, both of which have been comprehensively covered in a review by Lin and colleagues [24]. Following uptake, exogenous antigens are internalized into specialized organelles that are termed phagosomes for particulate/cell-associated antigens, or endosomes, for soluble protein antigens [25]. The “cytosolic pathway” involves escape of exogenous antigen from the endosome/phagosome into the cytosol for proteasomal degradation. Similar to direct presentation, this pathway is TAP dependent. However the mechanism allowing transfer of proteins into the cytosol, or the site at which peptides are loaded onto MHC class I molecules, is not fully known [26]. Several models have been proposed including (1) formation of a pore in the phagosome/endosome via the ER-associated degradation pathway (ERAD) translocon, Sec61, (2) ER-phagosome fusion for particulate antigens, and (3) ER-endosome fusion for soluble protein antigens [25, 26]. In contrast, the “vacuolar pathway” is TAP independent and suggests exogenous proteins are degraded into peptides by lysosomal proteases within the lumen of the phagosome (or endosome) [25, 26]. These peptides are then loaded onto recycling MHC class I molecules by peptide exchange. Of the two, the “cytosolic pathway” is believed to be the most physiologically relevant [24, 27]. However it may depend on the type of antigen, and the mechanism of uptake that decides the internal route to cross-presentation [25].

## 3. Dendritic Cell Subsets and Cross-Presentation of Tumour Antigen

DCs are believed to play a pivotal role in the initiation and programming of tumor-specific T-cell responses [14, 15, 28]. Indeed, DCs residing in the TDLN have been found to cross-present tumor antigen to naive CTL [3, 5, 8, 10]. Despite this, there is little information concerning the role of specific DC subsets in antitumor immune responses. Studies of DC cross-presentation in mouse models of viral infection have clearly demonstrated that different DC subsets display different capacities for cross-presenting antigens [29, 30]. For instance, the lymph node resident CD8*α* ^+^ DC appears to be specialized for cross-presentation of exogenous antigens to naïve CD8^+^ T cells [31–34], whereas migratory CD8*α* ^−^ DCs are required for presentation on MHC class II to CD4^+^ T cells [33, 35, 36]. However, recent findings have challenged this clear dichotomy, as cross-presentation by CD8*α* ^−^ DCs can be induced by triggering through TLR or FcR [33, 37–39], and CD8*α* ^−^ DCs are required for cross-presentation of antigen derived from *saccharomyces cerevisiae *[40]. Finally, CD8*α* ^−^CD103^+/-^ migratory DCs are able to cross-present viral antigen in the lung and skin [41–43] and may be the major cross-presenting subsets in these tissues. Therefore, the capacity of any DC subset to cross-present may depend on the nature of antigen encountered, and the state and location of the tissue where the DC resides, all of which may have important bearing on tumor antigen cross presentation. 

Considering the importance of DCs in determining the fate of tumor-specific T cells, it is perhaps not surprising that studies examining DC subset-specific cross-presentation of tumor antigen are now emerging. For instance, Murphy and colleagues demonstrated that CD8*α* ^+^ DCs were absolutely required for the generation of protective tumor-specific CTL using transgenic mice lacking expression of the transcription factor *Batf3 *[28]. Lack of *Batf3* in mice led to a selective loss of CD8*α* ^+^ DCs in the spleen and lymph nodes of these mice, whilst all other DC subsets remained intact. When these mice were challenged subcutaneously (s.c.) with syngeneic fibrosarcomas that are normally rapidly rejected in a CD8^+^ T-cell-dependent manner, tumors grew out [28]. This was associated with a failure to develop tumor-specific CTLs and hence tumor-infiltrating CD8^+^ T cells were reduced in these animals. Thus while CD8*α* ^+^ DCs appear crucial to the development of effective antitumor immunity, their role in the generation of ineffective CD8^+^ T cells associated with progressing tumors, has not been fully elucidated. In a murine model of melanoma, only CD8*α* ^+^ DCs isolated from TDLNs were able to cross-present the secreted tumor antigen, ovalbumin (OVA) [10]. In this case, generation of ineffective CTL was thought to be due to defective processing and presentation on MHC class II, rather than defective cross-presentation [10]. In contrast, we have recently shown that both CD8*α* ^+^ and CD8*α* ^−^ TDLN DCs were able to cross-present the membrane tumor antigen, hemagglutinin (HA) [5]. In this instance, the cross-presenting DC subsets differed significantly in their expression of the inhibitory B7 molecule, B7-DC, with abundant expression found only on the CD8*α* ^−^ subset. Thus B7-DC expression on the CD8*α* ^−^ DC subset might act to limit CTL generation because upon interaction with PD-1, B7DC has been shown to mediate suppression of CD8^+^ T cells [44]. 

The different conclusions drawn from these studies may be due to fundamental differences between the HA and OVA tumor models, secreted OVA versus membrane bound HA. A study by Burgdorf et al. demonstrated that mannose-receptor-mediated uptake of soluble OVA by DCs was required for cross-presentation to CD8^+^ T cells [45] and expression of the mannose receptor was observed exclusively on murine CD8*α* ^+^ DCs [46]. Thus, the fact that CD8*α* ^−^ DCs played an equivalent role in cross-presentation of HA tumor antigen in our system might reflect the cell-associated form in which the tumor antigen was delivered. This may have relevance for human cancer as in humans, several membrane associated tumor molecules have been shown to be antigenic, including gp100, tyrosinase, MUC-1, and carcinoembryonic antigen (CEA) in melanoma, mesothelioma and ovarian cancer, respectively [47–49]. The nature of the tumor antigen (i.e. membrane, nuclear, cytoplasmic, secreted) may therefore dictate the DC subsets required for cross-presentation and ultimately the CD8^+^ T-cell response that ensues. 

While much is known regarding the phenotype of cross-presenting DCs in mouse models of disease, their human counterpart has remained elusive. However, several recent studies have suggested that the human equivalent of mouse CD8*α* ^+^ DCs is the minor population of human blood DCs expressing blood dendritic cell antigen (BDCA)-3 (CD141) [50–55]. BDCA-3^+^ DCs share a similar transcriptional profile [55] and are known to express TLR3 [53], Clec9A [51, 54], Necl2 [53, 54], and XCR1 [50] like CD8*α* ^+^ DCs. In addition, they display an increased capacity for cross-presentation of soluble and cell-associated viral antigens [50, 53, 54]. However, due to their rarity in human blood (0.04%  ± 0%–30% SD, [50]), little is known regarding their role in the tumor setting. BDCA-3^+^ DC are known to infiltrate renal cell carcinoma [56] and are decreased in the peripheral blood of patients with hepatocellular carcinoma compared with healthy controls [57]. Further studies are required to determine the role of this subset in the cross-presentation of tumor antigen, and how they may be exploited for generation of a functional antitumor immune response.

## 4. Tumour Location and Cross-Presentation of Tumour Antigen

Solid tumors originate from diverse cell types and consequently can occur in many different locations in the body. Likewise the distribution, subset, and phenotype of DCs is known to differ between healthy peripheral tissues. In mice and man the lung, liver, kidney, and colonic mucosa are examples of sites that harbor different DCs subsets in different proportions, for example, murine lung DCs can be broadly subdivided into CD103^+^CD11b^−^ and CD103^−^CD11b^hi^ subsets that in the steady state are phenotypically and functionally immature [58, 59]. In contrast, colonic mucosa DCs are predominantly CD11b^+^ with a small proportion positive for CD103 [60]. Similarly human livers contain predominantly immature conventional BDCA-1^+^ DCs that produce IL-10 upon interaction with LPS [61]. In contrast pDC represent a greater fraction of the human kidney than conventional DC [62]. This most likely reflects the capacity of tissue resident DC to sample the local environment for innocuous and pathogenic antigen, yet it may also determine the generation of tumor-specific immunity at these sites. Murine studies have demonstrated that the antigen-presenting function of distinct DC subsets differs with the type and location of antigen exposure [41–43, 63–65]. A study by Chung et al. showed that anatomic location defined antigen presentation by DCs in response to soluble protein [65]. Following systemic injection of OVA, CD8*α* ^+^CD11b^−^ DCs were responsible for cross-presentation of OVA protein in the spleen whereas CD8*α* ^−^CD11b^+^ DCs cross-presented OVA in the mesenteric lymph nodes. Consistent with this result, CD8*α* ^−^CD11b^+^ DCs in the mesenteric LN were found to mediate cross-tolerance to intestinal antigens [66]. In response to innocuous inhaled antigen migratory CD103^+^ DC mediate cross-presentation in the thoracic lymph nodes, whereas CD103^−^ DC present antigen on MHC II to CD4^+^ T cells [43]. However upon influenza infection CD103^−^CD11b^hi^CD70^+^ DCs capture exogenous antigen in the lung and directly cross-prime CD8^+^ T cells in the draining lymph nodes [41]. Thus biological events in lymphoid tissue draining different sites may have direct relevance to tumor immunology because it is possible that the location in which a tumor is growing alters the response of the tumor-specific T cells. In support of this, IKDC were found in an experimental model of melanoma metastasis to be the dominant CD11c-expressing cells in the tumor bearing lung [67]. This subset developed preferentially in the lungs and was not detected in the adjacent lymph nodes [67]. In human cancer, analysis of peripheral blood DC revealed a disparity in the proportion of lymphoid to myeloid DC between patients with breast cancer and NSCLC, suggesting that the number of circulating DCs in cancer patients may correspond to the type of neoplasm [68]. Thus tumor growth at different locations may recruit specific DC subsets. Such knowledge may be crucial to the development of tumor immunotherapy's targeting-related tumors in different sites.

## 5. What Is the Source and Form of Tumour Antigen for Cross-Presentation?

According to current dogma, efficient priming of CTL against viral and self-antigens requires migration of DCs transporting antigens from peripheral tissues, followed by presentation in draining lymph nodes [69]. This is supported by evidence from viral systems whereby DCs migrating from the site of infection transport antigen to the TDLN for cross-presentation [70, 71]. DC infiltration of solid tumors is well documented in both tumor-bearing animals and patients [15, 18, 19, 72–74]. As such, generation of tumor specific responses might then be expected to involve migration of DCs from tumor tissue to the TDLN for cross-presentation of antigens. However, this model may not properly represent the generation of tumor-specific T-cell responses that occur in the steady state for several reasons; firstly, tumor growth is associated with a lack of proinflammatory signals and pathogen byproducts that may interact with tumor-resident DCs and induce their activation and subsequent migration to the TDLN [14]. Secondly, tumors produce a variety of immunosuppressive soluble factors that inhibit DC maturation and differentiation, leading to the recruitment and accumulation of immature DCs at the tumor site [14, 75]. Thirdly, several studies have shown that DC migration is impaired in the presence of a solid growing tumor [10, 76–78]. Finally, sentinel lymph node metastasis is a hallmark of human disease [79–84] and represents a mechanism whereby tumor antigen is delivered directly to the TDLN. While it is possible that DCs in the TDLN acquire antigen from metastatic tumor cells for cross-presentation to naïve T cells, the true form(s) of antigen required to induce an antitumor response *in vivo* is yet to be fully elucidated.

Tumor progression is associated with rapid proliferation of viable tumor cells and differing levels of tumor cell death in the form of apoptosis and necrosis. In addition, tumor cells are known to secrete soluble proteins [27, 85, 86] and antigen carrying exosomes [87, 88]. However the form of tumor antigen that is captured by DCs for cross-presentation is not known. Most of the information regarding the mechanisms operating in the capture, processing and cross-presentation of tumor antigens to CD8^+^ T cells have been generated from *in vitro* culture systems. A previous review of the literature highlighted the potential mechanisms for transfer of tumor antigen to DCs for cross-presentation [15] including; (1) phagocytosis of cell associated antigens, (2) pinocytosis/endocytosis of soluble antigen, (3) capture of soluble antigen bound to heat shock proteins (HSP), (4) gap junction transfer, (5) capture of exosomes, (6) “nibbling” of live tumor cell membranes, and (7) “cross-dressing” whereby DCs acquire peptide MHC complexes from contact with necrotic cells. While several of these mechanisms have been shown to mediate effective tumor antigen responses in the vaccination/treatment setting, their physiological relevance for generation of CD8^+^ T cells during normal tumor progression is not known, but could be vital for improving cancer treatment. 

Future studies should explore not only the form of tumor antigen required for effective cross-presentation but also any DC subset specificity. A study by Smyth et al. showed that splenic CD8*α* ^+^ and CD8*α* ^−^ DC subsets differ in their capacity to capture antigen for cross-presentation. Of note, CD8*α* ^−^ DCs induced CD8^+^ T-cell responses only when MHC:peptide complexes were acquired from neighboring cells, whereas CD8*α* ^+^ DC were more efficient at phagocytosis and cross-presentation [89]. While these mechanisms have not been explored in the context of antitumor immunity, they suggest that capture of tumor antigen from live tumor cells may favor cross-presentation by both DC subsets. In contrast, apoptotic cells have been shown to be preferentially captured by CD8*α* ^+^ DCs *in vivo *[90]. Since CD8*α* ^+^ DCs are required for rejection [28], therapies favoring capture of tumor antigen by this subset may facilitate more effective tumor-specific T-cell responses. Indeed, cellular destruction is known to enhance cross-presentation [91] and chemotherapy induced apoptosis of tumor cells has been shown to enhance tumor antigen cross-presentation [6]. As such, this concept may be explored using chemotherapy-induced apoptosis of tumor cells *in vivo* to determine whether there is a shift in the capacity of DC subsets to cross-present tumor antigen in the presence of apoptotic versus live tumor cells.

## 6. The Outcome of Cross-Presentation: Priming or Tolerance

Cross-presentation is required for the maintenance of peripheral tolerance (cross-tolerance) as well as the generation of CTL against infection (cross-priming). The balance between these two situations is believed to be mediated by the activation status of the cross-presenting DC [92]. Indeed, several studies have shown that the difference between tolerance and immunity is the presence or absence of appropriate inflammatory signals together with co-stimulation [92–94]. Such signals include pathogen-associated molecular patterns (PAMPs), derived from bacterial and viral infection, such as LPS and unmethylated CpG motifs that are recognized by specific receptors on DCs, including TLRs. Likewise, CD40-CD40 ligand (CD154) interaction with activated CD4^+^ T cells can result in DC activation. Together with soluble mediators such as type I IFNs, these interactions induce DC differentiation and activation characterized by increased surface expression of MHC molecules and costimulatory signals such as CD80, CD86, and CD70 a process often referred to as “licensing” [92]. Such signals are critical for the effective stimulation and enhanced survival of antigen-specific CD8^+^ T cells [95]. Conversely, cross-presentation by “unlicensed” or immature DCs stimulates an abortive CD8^+^ T-cell response culminating in deletion or anergy (tolerance), rather than the induction of effector CTL [94]. In both cancer patients and tumor-bearing animals, DCs infiltrating tumor tissue, or those found in local TDLNs bear an immature phenotype [14, 15]. Tumor beds have been shown to mediate this immune suppression by secretion of tumor-derived soluble factors such as IL-10, transforming growth factor-*β* (TGF-*β*), IL-6, vascular endothelial growth factor (VEGF), prostaglandin E-2 (PGE-2), and gangliosides that act to prevent DC differentiation and function [14, 75]. Similarly, altered levels of these cytokines in peripheral blood correlate with the presence of immature DC phenotypes [57] indicating that immunosuppression is not restricted to the local site. These immature DCs can induce tolerance through the generation of regulatory CD4^+^ T cells (Treg) [96], production of indoleamine 2,3-dioxygenase (IDO) [97–99] and expression of inhibitory B7 molecules, B7H1, and B7-DC, all of which act to suppress CD8^+^ T-cell activation and differentiation. Therefore, given the absence of inflammatory stimuli and local immunosuppression, cross-presentation of tumor antigens by immature DCs during normal tumor progression may result in CD8^+^ T-cell tolerance.

Given this, strategies aimed at activating the cross-presenting DC subset(s) and reducing immunosuppression show great promise for the treatment of cancer. Local and systemic administration of activating anti-CD40 antibody either alone or in conjunction with other therapies has been shown to alter the phenotype of DC subsets and augment tumor specific CD8^+^ T-cell responses *in vivo *[8, 100–103]. TLR agonists including polyI:C [104], CpG [74] and the TLR7/8 agonist Imiquimod [100, 105] have shown similar results. Of clinical relevance is a study by Stary et al. where treatment of basal cell carcinoma with Imiquimod promoted the recruitment of tumor infiltrating DCs expressing perforin and granzyme B, indicating that they may exert cytotoxic effects directly against tumor cells [105]. In addition to DC activating agents, several studies have shown that blocking interaction of B7 inhibitory molecules on DCs with their receptors on CD8^+^ T cells can promote productive antitumor responses *in vivo* [106–108], providing evidence that expression of these molecules by cross-presenting DCs may promote a defective T-cell response. However, it is not only new and emerging therapies that have garnered interest recently for their capacity to augment tumor-specific immune responses. In recent years, chemotherapy, used in the treatment of many primary cancers has been investigated not only for its role in tumor cell death, but also its ability to stimulate the immune system [6, 102, 109–115]. This immunoadjuvant effect of chemotherapy is thought to occur by altering the context of the dying tumor cell [109–111], increasing the amount of tumor antigen available for cross-presentation [6] or through side effects that stimulate the immune system [112, 116–118]. All of which may primarily rely on the capacity of DCs to capture, process and present tumor antigen from dying cells. A series of elegant experiments by the Zitvogel and Kroemer groups showed that DCs were exclusively required for the generation of protective immunity following injection with chemotherapy-treated tumor cells [110, 111]. Anthracyclin or oxaliplatin treatment induced (i) translocation of calreticulin (CRT) to the tumor cell surface, (ii) release of the TLR4 ligand high mobility group box 1 (HMGB1) protein, and (iii) release of ATP by dying tumor cells, all of which act in concert to promote IL-1*β* secretion by DCs and ultimately result in a protective tumor-specific CD8^+^ T-cell response [119]. The chemotherapeutic agent cyclophosphamide (CY) has been shown to enhance tumor specific immunity at low doses by specific depletion of CD4^+^CD25^+^ Tregs, [116, 120, 121]. High doses of CY induce lymphoablation and immunosuppression; however, this may set the stage for homeostatic proliferation, which may enhance any additional immunological intervention [121]. Furthermore, treatment of established solid tumors with a combination of CY and etoposide resulted in significant uric acid accumulation at the tumor site [122]. Uric acid has been shown to activate DCs and promote tumor rejection [122, 123]. In a similar vein, the chemotherapeutic agent gemcitabine can modulate the immune response to solid tumors by increasing the amount of antigen available for cross-presentation [124] and regulating tumor suppression by elimination of CD11b^+^Gr1^+^ myeloid-derived suppressor cells (MDSC) [112, 125, 126]. Importantly, gemcitabine has been shown to prime the host immune system for adjuvant immunotherapy. Treatment of tumour-bearing mice with activating anti-CD40 antibody following gemcitabine chemotherapy induced long-term cures in >80% of mice bearing malignant mesothelioma's (MM) [102]. This effect was not solely due to the debulking effects of the drug as surgical resection did not augment the effects of immunotherapy. Likewise gemcitabine chemotherapy prior to treatment with an adenovirus-expressing IFN-*β* led to regression of large established tumours [112]. While such effects are yet to be determined in humans, a study by Plate et al. demonstrated that following the initial infusion of gemcitabine in patients with pancreatic cancer the number of BDCA-1^+^ DCs in peripheral blood decreased, and this was countered by a reciprocal increase in the BDCA-3^+^ DC population [127]. BDCA-3^+^ DCs being a minor population in peripheral blood that are suggested to be the human equivalent of the murine CD8*α* ^+^ DC [50–55]. While numbers returned to normal during subsequent treatment this suggests that there may be a defined window during chemotherapy to apply immune modulating agents to favor cross-presentation of tumor antigens by particular subsets of DCs.

A knowledge of which DC subsets are present in tumors under basal and therapeutic conditions, along with information on their capacity to respond to various immune stimuli (such TLR agonists) is likely to guide the rationale design of future multimodality therapies.

## 7. Conclusions

DCs are a heterogeneous population of APCs that play an important role in the generation of effective CTL responses [30, 128]. Unfortunately, their function has been shown to be defective in tumor bearing animals and cancer patients [129], leading to an ineffective antitumor T-cell response, and ultimately, uncontrolled tumor growth and metastatic spread. While DCs have been studied extensively for their therapeutic or vaccine potential, their role during the effector phase of the antitumor response has not been fully elucidated. It has been demonstrated that DCs enriched from TDLNs are able to cross-present tumor antigen [3, 5, 8, 10] and importantly, that host dendritic cells are absolutely required for the generation of a protective CTL response leading to tumor regression [28]. Given this, it is not surprising that treatment strategies targeting endogenous DCs via CD40-CD40L interaction or TLR ligands have produced promising results. Therefore, a full understanding of the complex interactions between tumors and host DCs may reveal the reasons why the immune system fails to generate a protective antitumor response. 

Development of future therapies for cancer may be required to (1) alter the context of tumor antigen for presentation by host DCs, (2) sufficiently activate DCs in the local TDLN and at the tumor site, (3) target antigen to specific DC subsets, and (4) limit immunosuppressive mechanisms in the tumor microenvironment to enable reactivation of tumor-infiltrating T cells. Such outcomes will most likely be achieved through a combination therapies acting to induce tumor cell death and augment the host antitumor immune response.
